# Computational Methods for Modeling Aptamers and Designing Riboswitches

**DOI:** 10.3390/ijms18112442

**Published:** 2017-11-17

**Authors:** Sha Gong, Yanli Wang, Zhen Wang, Wenbing Zhang

**Affiliations:** 1 Hubei Key Laboratory of Economic Forest Germplasm Improvement and Resources Comprehensive Utilization, Hubei Collaborative Innovation Center for the Characteristic Resources Exploitation of Dabie Mountains, Huanggang Normal University, Huanggang 438000, China; shagong@hgnu.edu.cn; 2 Department of Physics, Wuhan University, Wuhan 430072, China; 13212746736@163.com (Y.W.), zhenwang@whu.edu.cn (Z.W.)

**Keywords:** riboswitch, aptamer, mRNA structure, gene regulation

## Abstract

Riboswitches, which are located within certain noncoding RNA region perform functions as genetic “switches”, regulating when and where genes are expressed in response to certain ligands. Understanding the numerous functions of riboswitches requires computation models to predict structures and structural changes of the aptamer domains. Although aptamers often form a complex structure, computational approaches, such as RNAComposer and Rosetta, have already been applied to model the tertiary (three-dimensional (3D)) structure for several aptamers. As structural changes in aptamers must be achieved within the certain time window for effective regulation, kinetics is another key point for understanding aptamer function in riboswitch-mediated gene regulation. The coarse-grained self-organized polymer (SOP) model using Langevin dynamics simulation has been successfully developed to investigate folding kinetics of aptamers, while their co-transcriptional folding kinetics can be modeled by the helix-based computational method and BarMap approach. Based on the known aptamers, the web server Riboswitch Calculator and other theoretical methods provide a new tool to design synthetic riboswitches. This review will represent an overview of these computational methods for modeling structure and kinetics of riboswitch aptamers and for designing riboswitches.

## 1. Introduction

**Figure 1:**
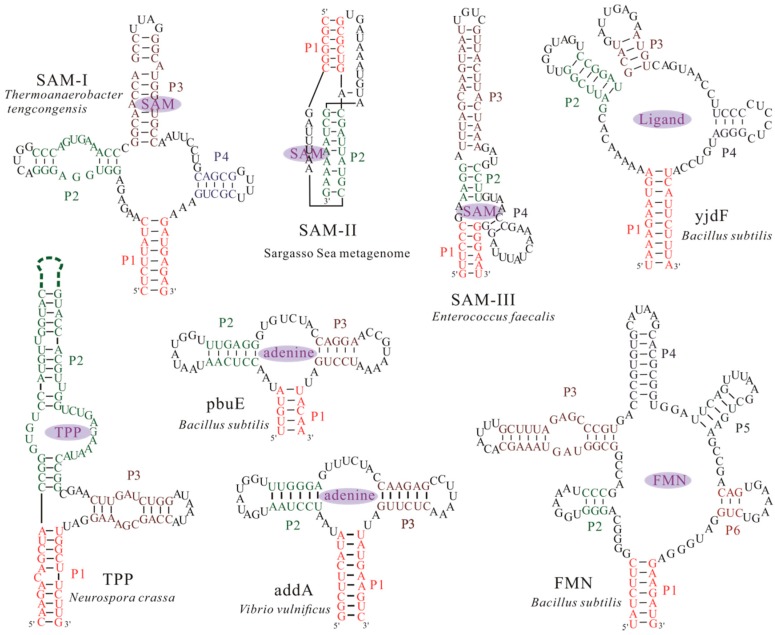
Structural models of aptamers from several extensively studied riboswitch candidates. The structures can bind their nature ligands, such as *S*-adenosylmethionine (SAM), thiamine pyrophosphate (TPP), adenine and flavin mononucleotide (FMN), to form a ligand bound conformation. Except the SAM-II riboswitch from the Sargasso Sea metagenome [23,24] and TPP riboswitch aptamer from *Neurospora crassa* [25], other aptamers are from bacteria. Nucleotides within helices P1, P2, P3, P4, P5, and P6 found within the bound aptamers, are colored differently. The dash line denotes the long helix region in the structure of TPP riboswitch aptamer.

Many noncoding RNAs (ncRNAs) have been found to bear important and diverse biological functions in all domains of life, including catalysis, protection of genomes, and regulation of cell activities [1,2,3]. This diversity in biological functions is attributed to the remarkable structure variety accommodated by RNAs. Riboswitches, which present a fundamental example of ncRNAs, are involved in cellular regulation through vast structural rearrangement in response to the intracellular physical signals, such as metabolites [1,4,5] and ions [6,7,8,9,10]. Among previously validated riboswitches, metabolite-specific riboswitches are the most widespread and the nature of their ligands is well defined in most cases, except for several “riboswitch-like”, presumably *cis*-acting, RNA structures for which no ligand has been found yet [11,12]. In order to function, most riboswitches usually consist of two domains: a conserved aptamer domain that is responsible for ligand binding, and an expression platform that converts changes in the aptamer domain into changes in gene expression. In contrast to these riboswitches, the S_MK_ (SAM-III) riboswitch is the one that can use single domain for both ligand binding and gene regulation [13,14,15]. Aptamer domains, which are typically 35~200 nucleotides in length [16,17,18], often form a ligand-binding pocket (the aptamer structure) to bind the ligands with high specificity. To date, according to the aptamer structures (Figure 1), more than 30 riboswitch classes have been found in all three kingdoms of life [19,20,21]. Due to their specificity and function as genetic regulators, riboswitches represent a novel class of molecular target for developing antibiotics and chemical tools [22]. Thus, a comprehensive understanding of riboswitches is important to facilitate the design for riboswitch-targeted drug, molecular robotics, and new molecular sensors.

**Figure 2:**
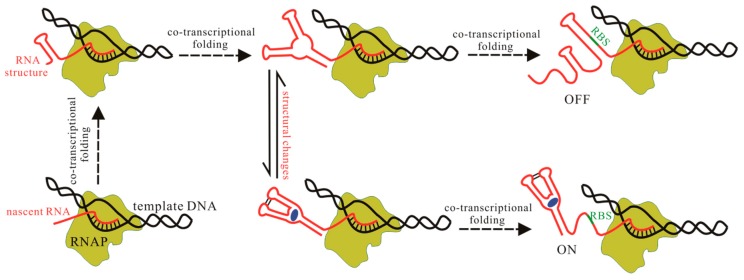
Schematic representation of riboswitch behaviors in cells. Nascent chain of the riboswitch and the ligand are colored red and blue respectively. The green line denotes the ribosome binding site (RBS) and RNA polymerase (RNAP) is denoted by yellow-green. The structure, structural changes, and co-transcriptional folding should be investigated for fully understanding the regulation mechanisms of riboswitches.

The signal-dependent conformational shifts of riboswitches, usually between two distinct functional states, i.e., ligand bound state and unbound state, regulate the downstream gene expression (Figure 2). One of the alternative states serves as the genetic off state (OFF state) by forming an intrinsic terminator hairpin or a repression stem to repress gene expression [26,27,28]. The other state acts as the genetic on state (ON state), which induces gene expression by preventing the formation of these regulatory elements. Riboswitches can also regulate RNA splicing by controlling the structural flexibility near the relevant splice site [25]. During the regulatory activities, one of the two structures is adopted by riboswitches, depending on whether the aptamer domain can form the pocket and bind its ligand on time or not. Therefore, to investigate the regulation mechanism of these functional ncRNAs, one of the major challenges is the information of the aptamer structure (Figure 2). In contrast to proteins, a much smaller number of RNA structures have been solved by using the traditional experimental methods [29], such as nuclear magnetic resonance (NMR) spectroscopy and X-ray crystallography [15,30]. Sensitive X-ray crystallography must take special care to avoid RNA aggregation and misfolding prior to crystal formation, and NMR experiments easily suffer from poor long-range correlations for RNA. These create a great demand to obtain information of RNA structures by using theoretical approaches. Up to now, many packages and methods have been developed to predict RNA secondary structure (two-dimensional (2D)) [31,32,33], as well as the three-dimensional (3D) structure for small RNAs [32,33,34,35,36,37,38]. These methods can quickly produce structure for a given RNA based on the input data. Some computational methods, such as RNAComposer and Rosetta [34,35,39], have been successfully applied to modeling 3D structure for several complex aptamers. As classical experimental methods may be limited in applicability to RNA [40], these theoretical approaches are likely to circumvent the bottleneck from experimental methods.

Another scientific challenge for the elucidation of the riboswitch function is to model aptamer kinetics, which is intrinsic to folding and conformational switching. Traditional molecular dynamics (MD) simulations [41,42], are able to provide a direct link between structure and dynamics. Nonetheless, due to the extreme complexity of force fields, a large number of atoms and a high number of degrees of freedom in RNA molecules, the detailed all of the atom simulations are difficult to produce trajectories in the time frame relevant for function of aptamers, which usually need to undergo large structural changes for purposes. Coarse-grained structural model with a less exhaustive representation is therefore particularly efficient to deal with these problems [43,44]. For example, the folding kinetics of *pbuE* [45], *addA*, and S_MK_ riboswitch aptamers [43,46], have already been studied by using the coarse-grained SOP model. Another efficient alternative based on coarse-grained model is to investigate the kinetics of RNA 2D structure [47,48], which can also capture enough detail to understand the functions of aptamers.

Like protein, which can fold as soon as the N-terminal part emerges from the ribosome (co-translational folding) [49], nascent RNA also folds spontaneously as the nucleotides are synthesized by RNA polymerase in living cells (co-transcriptional folding) [50]. As riboswitches fold co-transcriptionally, folding patterns of the aptamer domain can direct the folding of the downstream expression platform. In fact, since many riboswitches regulate transcription, ligand binding can only occur during transcription for these to control gene expression [26,51,52,53]. Hence, the co-transcriptional folding kinetics is crucial for understanding the intracellular function of aptamers. Besides the optical-trapping assay and other experimental approaches [28,50,54], the kinetic Monte Carlo (MC) method was used to study the co-transcriptional folding of riboswitches [55], but it only considered the base pairing interactions closed in the native structures. By combining the master equation and the free energy landscape, BarMap and the helix-based computational methods have been applied to modeling the co-transcriptional folding behaviors for several riboswitches [56,57,58,59,60]. The results suggest that the aptamer domain folds into the pocket structure as soon as the nucleotides are transcribed [57,58], while the riboswitch without a separate aptamer domain is more likely to form an alternative structure instead of the pocket during the transcription [56]. These computation models, which can predict stable and metastable structures, kinetics, and transition states, bridge the gap in understanding the relationship between the structure and biological function of aptamers. Furthermore, computational RNA design has also made a great progress to construct synthetic riboswitches by using different strategies [61,62]. Here, we will provide a collection of these computational methods for modeling the structure and kinetics of aptamers, and for designing riboswitches.

## 2. Computational Method for Predicting Aptamer Structures

Since the structure of RNA determines its biological function, a complete understanding of the aptamer structure is the necessary prerequisite to understand the riboswitch-mediated regulation processes in the cell. RNAs fold into complex structures; the linear ribonucleotide sequence is the determinant of base-pairing interactions (2D structure), which in turn, determines the spatial shape (3D structure). Since most computational methods use the input of 2D structure to produce the RNA 3D structure [29,32,63], the precise prediction of 2D structure becomes more and more important. Early computational approaches for predicting RNA 2D structure (e.g., RNAfold [64]), only find the structure with the lowest free energy for a given RNA. As the functional structure may not be the one with the lowest energy, methods such as *mfold* and RNAsubopt [31,65] are developed to predict a set of low-energy structures. Other prediction methods, such as PPfold and RNAalifold [66,67], are based on evolutionary considerations. For a given aptamer, these methods can quickly produce 2D structure and the free energy. The methods for the modeling structure do not consist of a process that assumes co-transcriptional folding. Recently, RNA 2D structure prediction has been reinforced by incorporating the constraints from the experiments [65].

### 2.1. RNAComposer

**Figure 3:**
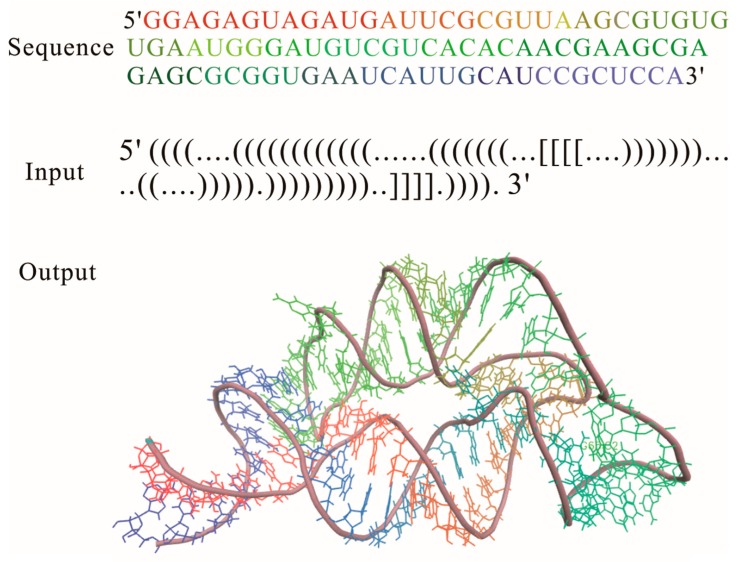
Three-dimensional (3D) structure for THF riboswitch aptamer (PDB code: 3SD3) predicted by using RNAComposer program online.

RNA 2D structure is a crucial step in the functional characterization, but a thorough understanding of aptamer functions depends critically on the 3D structure, which is the key determinant of their interactions with ions and other molecules in cell. Based on the 2D structure tree graph representation and homology of structural elements, the RNAComposer method (Table 1) was developed to automatically predict the 3D structure for large RNAs [29]. As a knowledge-based method, it uses RNA sequence and 2D structure topology in dot-bracket notation as an input for 3D structure prediction (Figure 3). In this notation [64], unpaired nucleotides and the nucleotides that are involved in base pairs are represented by dots and brackets, respectively; square brackets and curly brackets refer to first-order pseudoknots and higher order structures, respectively. Although the input RNA 2D structure can be obtained by using the methods that are incorporated within the RNAComposer system: RNAfold, RNAstructure, and Contrafold, experimentally adjusted 2D structure is able to largely improve quality of the prediction [39].

The input RNA 2D structure first is divided into stems, loops, and single strands in the program. 3D structure elements corresponding to these fragmentations are searched within the structure elements dictionary, which is tailored from the RNA FRABASE database and consists of a 3D structural element with good structural properties [68]. After the searching process, the 3D elements whose heavy-atom root mean square deviation (RMSD) is lower than 1.0 Å relative to the parent PDB structure, are selected according to the 2D structure topology, sequence similarity, and so on. By merging the selected 3D structure elements, the initial RNA 3D structure is obtained, and then refined by the energy minimization in the torsion angle space and Cartesian atom coordinate space to get the final 3D structure. RNAComposer has been used to accurately build the 3D structure of several complex riboswitch families [39], such as FMN riboswitch aptamer, TPP, and purine riboswitch aptamers. It is offered at two sites: http://rnacomposer.ibch.poznan.pl and http://rnacomposer.cs.put.poznan.pl. By typing the 2D structure of THI riboswitch aptamer in Figure 3 on the website [69], the related 3D structure will be released in PDB formatted file to users within 10 s. RNAComposer is automated, efficient, especially suited for RNA 3D structure prediction of large RNAs, but it highly depends on the 3D structural elemental dictionary, and the applicability of the method is limited to RNAs with a few complex kink turn motifs.

### 2.2. Rosetta and Discrete Molecular Dynamics

RNAComposer is motif library-based, while Rosetta is a fragment-based method that is available online [35]. In the Rosetta approach, according to the RNA 2D structure and the experimental proximity mapping data, low-resolution models are generated by using Fragment Assembly of RNA (FARNA) [34], where models are assembled using RNA fragments from a crystallographic database via a MC algorithm. The Rosetta all-atom energy function is then used to minimize a small number of the low-resolution models with the lowest Rosetta energy scores. To select a representative set of 3D models, the largest and lowest-energy subsets of models that fall within a certain RMSD threshold of each other are collected to reflect the native fold of the RNA. Taking the aptamer from *Fusobacterium nucleatum* double glycine riboswitch as an example, the Rosetta 3D modeling can give a similar prediction as RMdetect and JAR3D [70,71,72,73].

Also, starting from 2D structures, a hierarchical computational approach (RAGTOP) was modified to predict 10 representative riboswitch aptamers with diverse structural features [74], by combining the coarse-grained graph sampling approach [76]. Through integration of computational and experimental methods, a three-bead coarse-grained model of RNA for discrete molecular dynamics simulation have gotten precise 3D structure predictions for the M-box riboswitch and TPP riboswitch aptamers [77], and for RNA in the range of a few hundred nucleotides [40]. In this coarse-grained model, each RNA nucleotide is represented by three beads corresponding to the base, sugar, and phosphate groups. The potential terms includes bonded, non-bonded interactions, and additional potential terms based on the experimental hydroxyl radical probing data. This method uses RNA sequences and base pairing as inputs to generate structures, and then applies replica exchange simulations with the potential to find a representative structure. Based on this method, the web server iFoldRNA was created for prediction of RNA 3D structure [75].

The 3D structures of aptamers modeled by these approaches suggest that the aptamer domains often form a compact structure involving many complicated tertiary interactions. Since the entire prediction of these approaches depends on the input data, the correct 2D structure is critical for the accurate 3D structure modeling. Experimental data provides powerful constraints to reinforce 2D structure prediction, but these methods currently can only achieve subhelix-resolution accuracy or near-atomic accuracy for RNAs.

## 3. Computation Model to Characterize Structural Changes in Aptamers

A key event in the biological function of riboswitches is the structural change within the aptamer domain. This change can lead to a change of the folding pattern within the expression platform, thereby directly modulating the gene expression. For effective flipping of riboswitches, the structural change of aptamers must be achieved within the certain time window. Therefore, characterizing the structural changes of aptamers is also important for fully understanding their function in the cell. The conventional all atom MD simulation has been widely used to describe the time-dependent motions of biological molecules [78]. However, even though modern parallelization of MD simulation is able to model trajectories on the order of milliseconds, it still fails to address the majority of biological processes, including folding or unfolding of aptamers that occur on much longer timescales (in seconds). In order to model time-dependent structural changes of aptamers, an effective solution is to use a coarse-grained structural model.

### 3.1. The Master Equation Approach

The master equation approach or kinetic MC method based on coarse-grained system [79,80,81], namely RNA 2D structure, is advantageous for accessing behaviors at long time scales, even minutes or hours [80]. In the master equation approach, structural changes are usually modeled on the RNA energy landscape, which specifies the conformation space and the transition rates between conformations. For a given RNA, the conformation space is sampled by all of the possible 2D structures that are constructed by using the formation or disruption of an entire helix as the elementary step to allow for large structural changes. Their free energies can be calculated using the Turner energy parameters [82,83]. The transition rates between states are obtained based on the free energy landscape analysis [48,80,81]: (1) formation; (2) disruption of a helix; and, (3) helix formation with concomitant partial melting of a competing helix. With these key concepts, the folding process can be described by the master equation:

(1) $$dp_{i}(t)/dt=\sum_{i\neq i}[k_{j\to i}p_{j}(t)-k_{i\to j}p_{i}(t)]$$

where $p_{i}(t)$ is the population of state *i* at time *t* and $k_{i\to j}$ is the transition rate from state *i* to state *j*. This computational method which integrates RNA 2D structure with the static energy landscape, provides a basic idea to predict the folding kinetics of the Hepatitis delta virus ribozyme and S_MK_ riboswitch [56,80].

### 3.2. Coarse-Grained SOP Model

Besides these approaches, the coarse-grained SOP model using Langevin dynamics simulation can also be used to study the kinetics of RNA molecules by characterizing the folding landscape [43,45,46]. Actually, many complex biological processes, such as unfolding and refolding of various RNA and proteins [44,84,85], are described with great success by using the SOP model. In this coarse-grained model, each nucleotide, as well as the ligand, is represented as a single site. The total potential energy of the bound aptamer is

(2) $$V_{T}=V_{\mathrm{APT}}+V_{\mathrm{APT}-L}$$

where $V_{\mathrm{APT}}$ is the energy function of the aptamer; and, $V_{\mathrm{APT}-L}$ is the interaction between the ligand and the aptamer. The dynamics of the system can be simulated by using Brownian dynamics or the Langevin equation in the overdamped limit. During the force ramp simulation, the 5′-end of RNA is attached to a spring pulled with a constant speed, while the 3′-end is fixed. The free energy profiles are obtained using

(3) $$G_{z}=-k_{B}T\ln P(z)$$

where $k_{B}$ and T are the Boltzmann constant and temperature, respectively; and, the probability P(z) of the extension between *z* and *z + dz*, is calculated from the folding trajectories. Based on the theory of mean first-passage times [84], the transition rate between two folding states can be calculated from the time traces of the extension.

The kinetics of the SAM-III and *addA* riboswitch aptamers have been successfully studied with this approach [43,46]. As the crystal structure of *pbuE* riboswitch aptamer is not available, its atomic structure is produced via conformational sampling with MD by substituting the sequence of *pbuE* aptamer into the crystal structure of *addA* aptamer (PDB code: 1Y26), when considering the structural similarity between the two aptamers. But despite this structural similarity, their folding behaviors are different in the pulling simulation [43,45]. In *addA* riboswitch aptamer (Figure 1), the unfolding occurs in the order of F→P2|P3→P3→U, while the unfolding order of *pbuE* aptamer is F→P2|P3→P2→U, where F and U denote the fully folded state and unfolded state, respectively; Pi is the hairpin structure with helix Pi; P2|P3 denotes the state with helices P2 and P3. The different unfolding order of P2 and P3 in the two aptamers suggests that the riboswitches carry out different regulatory activities in bacteria, even though they belong to the same class. Helix P3 in *pbuE* riboswitch is unfolded ahead of helix P2, because of its unstability. This can explain why helix P3 is disrupted in OFF state of *pbuE* riboswitch but keeps folded in that of *addA* riboswitch [28,86]. Due to this greater conformational change in *pbuE* riboswitch, its OFF state can hardly transit to the aptamer structure, implying an irreversible kinetic riboswitch [57]. On the contrary, *addA* riboswitch is able to quickly reach equilibrium between the OFF state and the aptamer structure, which is consistent with a reversible thermodynamic switch [28]. The different unfolding kinetics of the aptamers under force thus can provide the information of their function.

## 4. Methods to Predict the Structure Transitions during Transcription

RNA folding occurs in two different ways [87]: (i) folding after synthesis of the entire RNA molecule (refolding); and, (ii) sequential folding occurs during transcription, namely co-transcriptional folding. In vivo, most RNAs fold co-transcriptionally, due to the sequential nature of RNA synthesis. The sequential folding during transcription is crucial for riboswitches, especially the kinetic riboswitches, to exert their regulation. Since kinetic switches are trapped in one state depending on whether the trigger is present at the time of folding, the mechanism of their function can only be understood in the context of co-transcriptional folding. For example, the full length *pbuE* riboswitch quickly folds to OFF state, which hinders adenine binding, while the aptamer structure that is responsible for ligand binding is not observed [53,57]. However, as nucleotides of the aptamer domain are transcribed first, the sequential folding may allow for the aptamer to form the pocket and bind to the ligand before formation of OFF state during transcription. Thus, the co-transcriptional folding kinetics of aptamers during transcription plays an important role for understanding their function in living cells.

In the case of co-transcriptional folding, as the RNA chain grows, the whole transcription process can be divided into a series of transcription steps [48,57,59], with each corresponding to adding one nucleotide. RNA folding kinetics within each step is modeled on the energy landscape as a certain RNA chain. But a link between two consecutive steps should be constructed due to the sequential folding in the transcription context. Based on this idea, the BarMap approach and helix-based computational approach have been developed to investigate the folding behaviors of several riboswitches under different transcription conditions [56,57,58,88].

### 4.1. BarMap

The BarMap approach integrates RNA 2D structure with the dynamic energy landscape to explore co-transcriptional folding kinetics [59]. The main idea of this approach is to model RNA kinetics on individual landscapes, where external triggers are considered as discrete changes. For successive kinetic simulations, a map between states of adjacent landscapes is computed to define the transfer of population densities.

**Figure 4:**
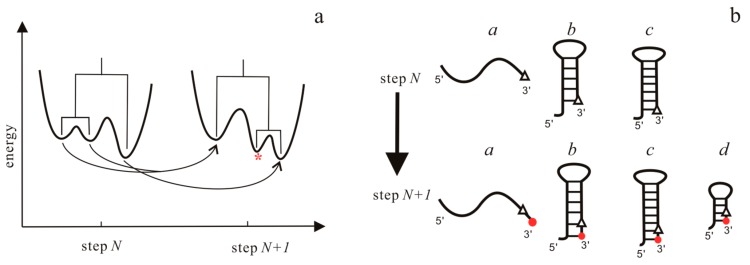
Relationship of landscapes in the BarMap approach (**a**) and structures in the helix-based computational method (**b**) between two adjacent steps. In (**a**), there are three types of events in landscapes: (i) A one-to-one correspondence between two minima (right); (ii) A two-to-one correspondence (left); and, (iii) a new local minima (marked by *) appears in the landscape at step *N* + 1. In (**b**), the triangles and red dots denote the newly transcribed nucleotide at step *N* and *N* + 1, respectively. For structures belonging to the first three types, their initial population at step *N +* 1 “directly inherit” from step *N*, while for type *d*, the initial population of the structure at step *N* + 1 is zero.

In the case of transcription, the folding is treated as a process on a time-varying landscape in this approach. For each RNA elongation step, an energy landscape is first computed using the barriers program, which can simulate RNA folding kinetics [89,90]. Then, BarMap constructs a map between the landscapes of two successive steps, as shown in Figure 4a. According to this map, the final population at the previous step can be mapped to the initial population on the landscape at the current step. Finally, starting with the first landscape with more than one state, RNA folding kinetics can be simulated by the treekin program [59], which integrates the master equation for arbitrary times through calculating the matrix exponential. The amount of time *t* on a particular landscape corresponds to the elongation time of the polymerase. Co-transcriptional folding behaviors of RNAs can be obtained from the relationship between the population density and the transcription step that is given by the approach.

The BarMap approach has been used to study the RNA thermometer, RNA refolding during pore translocation, and co-transcriptional folding [59,88]. The effects of transcriptional pause sites, transcription rates and ligand concentrations, can easily be included in the approach by specifying the amount of time *t* and changing the binding energy added to all of the ligand-competent states, respectively. Using the example of a recently designed theophylline-dependent RS10 riboswitch, the BarMap approach predicts the folding behaviors in good agreement with experimental observations [88].

### 4.2. The Helix-Based Computational Method

In helix-based computation model [60], the folding time window for each RNA elongation step also depends on the time required for RNAP to transcribe the relevant nucleotide. At each step, the population kinetics is calculated in the same manner as the refolding kinetics that are calculated in the master equation approach [48,60]: first, the conformation space is generated and the transition rates are calculated; then, the population relaxation within the folding time window is described with the master equation, where the initial population at the current step is determined by the folding results of the previous step. Based on possible structural changes as the RNA chain is elongated by one nucleotide, a link between the initial population distribution at the current step and the final population distribution at the previous step is constructed in Figure 4b. Like the BarMap approach, the effects of the transcription speed, transcriptional pause, and ligand concentration can be mimicked by modifying the folding time window for each step and the binding energy in this method.

**Figure 5:**
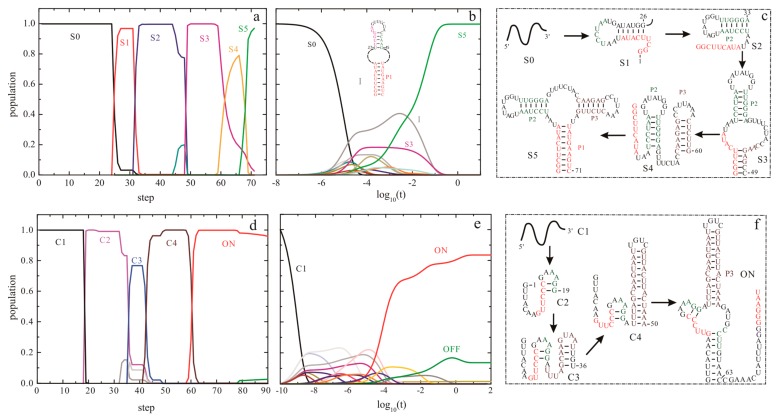
The co-transcriptional folding kinetics (**a**,**d**) and refolding kinetics (**b**,**e**) of *addA* riboswitch aptamer (up panel) and S_MK_ riboswitch (bottom panel). The transcription rates for *addA* aptamer and S_MK_ riboswitch are 25 nt/s and 50 nt/s, respectively, and the main intermediate states formed during the transcription are shown in (**c**,**f**). The dash dot lines in (**b**) for *addA* riboswitch aptamer denote the unpaired nucleotides within the large inter-loop. OFF state of S_MK_ riboswitch is the structure that is responsible for SAM binding shown in Figure 1.

The helix-based computational method has been used to explore the regulation mechanisms of several well-studied riboswitches, such as *pbuE* [57], S_MK_ [56], *metF*, and *yitJ* riboswitches [58]. The results show an excellent agreement of predicted trajectories with that from experiments and other methods [50,55,91]. The folding behavior of *addA* riboswitch aptamer, as shown in Figure 5, suggests that as the chain grows, the nascent RNA chain folds through a series of discrete intermediate states (from S0 to S5). When the first 25th nucleotide is transcribed, the open RNA chain S0 begins to form structure S1, which is replaced by S2 from step on 32. As the 49th nucleotide is free to form structures, S3 is formed and occupies most of the population till the 59th step. From step on 59, S3 begins to transit to S4. When helix P1 can be nucleated, the chain folds into S5.

This aptamer domain can fold into the pocket S5 as soon as the relevant nucleotides are transcribed (Figure 5a,d), which has also been found in *pbuE* [57], *yitJ*, and *metF* riboswitches [58]. During the refolding process (Figure 5b,e), the entire molecule folds into S5 mainly through structure I and S3. Although the refolding pathway is different from the co-transcriptional folding pathway, the aptamer domain also can form S5 within 0.1 s, implying that the aptamer domain is highly evolved. In contrast to these riboswitch aptamers, the SAM-III riboswitch, which utilizes a single domain to exert functions, quickly folds to ON state instead of the pocket (OFF) structure (as shown in Figure 1) under both the transcription context and the refolding condition (Figure 5) [56]. This thermodynamic switch is not sensitive to co-transcriptional folding kinetics, so it can be understood by the equilibrium properties. The co-transcriptional folding pathway of *addA* aptamer is similar to that of the *pbuE* aptamer [57], possibly because of the conservation within the aptamer domains. For many riboswitches, since the expression platforms are required to form a terminator or a repression stem [1,4], their sequences could be helpful to decipher the different folding intermediates as well.

Previous studies suggest that transcriptional pause plays a key role for riboswitches and other RNAs to exert function [92,93,94,95]. The major transcriptional pause sites found within several riboswitches are located immediately after the aptamer domains [51,86,96]. As the time window that is allowed for ligands binding is limited during transcription, the pauses in these regions can give the aptamers extra time to bind to the ligands but prevent the unbound functional states from being formed. Their effects have been assessed by the helix-based computational method and experimental approaches [51,57,58,93]. The results from the helix-based computational method suggest that removing the pause sites leads to a demand for an even higher ligand concentration to trigger the switch [57,58].

The good agreement of the results from these theoretical approaches with the experiments and other methods implies that they provide a reliable tool to understand the function of aptamers in the riboswitch-mediated gene regulation. However, all of these approaches based on 2D structure prediction ignore tertiary interactions and the effects of ions. Although the ligand binding can be mimicked by modifying the binding energy from experiments, the 2D structure model still cannot precisely predict the ligand–RNA interactions. Hence, there is a significant requirement in incorporating these factors to fully understand the function of riboswitches.

## 5. Computation Design for Synthetic Riboswitches

Artificial riboswitches established recently demonstrate that they can be used as a new tool for the drug-regulated expression of viral genes [97]. To design artificial riboswitches or other RNA devices for industrial and medical applications, different computation strategies have been developed over the years [62,98,99,100,101,102]. Based on a biophysical model of translation initiation, a web server called Riboswitch Calculator can be used to design synthetic riboswitches form various RNA aptamers [62]. In this model, a riboswitch is considered as a long mRNA molecule. The interactions of RNA–RNA, RNA–ribosome and ligand–RNA, control the translation initiation rate, which can be calculated as:

(4) $$r=\exp(-\beta \Delta G_{\mathrm{total}})$$

where β, $\Delta G_{\mathrm{total}}$ are the apparent Boltzmann coefficient and total energy change between mRNA and initiating 30S ribosomal subunit. The energies of RNA folding and the ribosome binding to mRNA are calculated using the ViennaRNA suite and RBS Calculator [64,103], respectively.

Riboswitch Calculator provides a useful tool to design translation regulating riboswitches. The four inputs are necessary for the design: the sequence and structural constraint of an aptamer, the ligand binding energy, and the protein coding section. These inputs are used by the optimization algorithm to generate an initial set of riboswitch candidates. Rounds of random mutations, evaluation, selection, and recombination are performed using these candidates. The biophysical model calculations are employed for the fitness evaluation to select the candidates that meet the objective function requirements, such as reaching targeted translation rates in OFF and ON states. The selected candidates will be sent back for the next round to find the riboswitch candidates with the highest fitness.

Transcription regulating riboswitches can also be designed by using the computational method [61]. The design algorithm used the aptamer with the known sequence and secondary structure. When considering the terminators always with a minimal size and the U stretch, sequences with lengths between 6 and 20 nucleotides are randomly generated to create a spacer database. The following step is to generate the sequences that are complementary to the subsequences of the 3′ part of the given aptamer. With these terms, a riboswitch candidate can be created by concatenating the aptamer, a spacer, a complementary sequence, and the U stretch. Using the RNAfold program [64], these candidates are evaluated by the folding simulations to select the elite members. A theophylline-specific riboswitch that is generated by this approach can activate transcription on binding of the target ligand [61].

These computational methods can be applied to constructing synthetic riboswitches, but the evaluation of synthetic riboswitch candidates is based on thermodynamic calculations in both of the approaches. Besides the above approaches, other methods that use different strategies are developed to design RNA devices recently [99,100,101,104]. It is known that co-transcriptional folding kinetics is fundamental to the action of functional RNAs in cell. However, it is not well incorporated into these methods, possibly because of the complexity of co-transcriptional folding. Although the transcription context may pose a serious challenge in the quest for designing RNA devices, these methods have made a great progress in the development of computational methods for designing RNA devices.

## 6. Conclusions

Since functions of biological molecules are determined by the formed structures, precise structure precondition is crucial to a complete understanding of many RNA-mediated processes, such as the regulation activities of riboswitches. Two domains of riboswitches, namely, the aptamer domain and the expression platform, often share nucleotides. The aptamer domain usually needs to form a unique ligand-binding pocket for specifically binding its ligand, which in turn, locks the conformation of the aptamer domain and directs the folding of the downstream expression platform. However, if the aptamer fails to bind its ligand, part of the nucleotides within the aptamer domain will pair with the nucleotides within the expression platform to form an alternative structure. For the riboswitches with one single domain, conformational change induced by the ligand binding would result in the formation of the structure that is different from the unbound functional state.

As structural changes within the aptamer domain produce a substantial change in the gene expression, it therefore demands to characterize structure and kinetics of aptamers for a thorough understanding of the riboswitch-mediated regulation. Given the fact that most aptamers have been extensively studied experimentally, yet they are not well characterized in some aspects. In the last several decades, many computational methods have been developed to investigate aptamers. One primary concern for aptamers is to precisely model the structure that is responsible for ligand recognizing and binding. Effective approaches, such as the motif library-based and fragment-based methods, have made significant progress in 3D structure prediction by integrating knowledge-based algorithm with experimental data or physics-based model. However, 3D modeling for aptamers and other large RNAs has not achieved a consistent atomic accuracy and the data may not enable statistical meta-analysis currently. Most of them are not suitable for predicting the 3D structure for highly complex RNAs. Furthermore, RNA chaperones may play a role in RNA folding [87,105,106], which are not considered in the model. High quality 3D structure prediction may require more experimental mapping data and significant human insight to build accurate models. Further advancements may hold great promise for making progress toward the goal.

Another important issue for aptamers comes from describing their great structural changes. Modern parallelization of MD simulation largely improves the computation efficiency, but it still cannot address the structural rearrangements of aptamers that occur on a seconds’ timescale. The coarse-grained SOP model and other computational methods have been applied to characterizing the kinetics of several riboswitch aptamers with great success. These demonstrate that the coarse-grained structural model, which enhances the conformation sampling, can be used to study the large scale conformational fluctuations of RNAs.

Also based on the coarse-grained structural model, a number of computational methods, such as RNAkinetics [107], Kinefold [108], and COFOLD [47] are developed to simulate co-transcriptional folding pathways of mRNAs from the early 1980s when key experiments show that structure formation happens co-transcriptionally [109]. In order to investigate the riboswitch-mediated regulation mechanisms, ligand binding should be taken into account. By incorporating the effect of ligand binding, the BarMap package and helix-based computational method have been successfully used to predict the co-transcriptional folding for several riboswitches. The application of these approaches in riboswitches implies that the 2D structure model can capture enough details of their behaviors in living cells.

Since conformational changes in aptamers can be induced by ligands, some other theoretical approaches focus on the prediction about interactions between ligands and aptamers or other features [110,111,112]. In recent years, computational methods for designing riboswitches have also made excellent progress in synthetic biology. As discussed above, several notable limitations of these computational methods still exist. Continuing developments in computational and hybrid methods are expected to overcome these limitations.

## Figures and Tables

**Table 1:** Methods which have been used for modeling 3D structure for aptamers.

Methods	Description	Availability	Reference
RNAComposer	A motif library-based method that uses the dictionary tailored from RNA FRABASED database to build initial 3D structure.	Web server	[39]
Rosetta	A fragment-based method that uses FARFAR optimizes RNA conformations in the context of a physically realistic energy function.	Local installation	[72]
RMdetect	A bioinformatics tool for identifying known 3D structural modules on genomic sequences.	Local installation	[73]
JAR3D	Scoring sequences to motif groups based on sequences’ ability to form the same pattern of interactions in motif.	Web server	[70]
RAGTOP	Predicting RNA topologies by a coarse-grained sampling of 3D graphs guided by statistical knowledge-based potentials.	Not available online	[74]
iFoldRNA	Incorporating SHAPE into discrete molecular dynamics to predict RNA structure.	Web server	[75]

## Abbreviations

ncRNA	noncoding RNA
SOP	Self-Organized Polymer
MC	Monte Carlo
FMN	Flavin Mononucleotide
SAM	*S*-adenosylmethionine
TPP	Thiamine Pyrophosphate
NMR	Nuclear Magnetic Resonance
MD	Molecular Dynamics
RNAP	RNA polymerase
RMSD	Root Mean Square Deviation
FARNA	Fragment Assembly of RNA
RAGTOP	RNA-As-Graph-Topologies
